# Delirium screening with 4AT in patients aged 65 years and older admitted to the Emergency Department with suspected sepsis: a prospective cohort study

**DOI:** 10.1007/s41999-021-00558-5

**Published:** 2021-10-08

**Authors:** Marius Myrstad, Kanika Kuwelker, Sigurd Haakonsen, Therese Valebjørg, Nina Langeland, Bård Reiakvam Kittang, Guri Hagberg, Bjørn Erik Neerland, Marit Stordal Bakken

**Affiliations:** 1 Department of Internal Medicine, Bærum Hospital Vestre Viken Hospital Trust, 1346 Gjettum, Norway; 2 Department of Medical Research, Bærum Hospital Vestre Viken Hospital Trust, 1346 Gjettum, Norway; 3 Department of Medicine, Haraldsplass Deaconess Hospital, Bergen, Norway; 4 Department of Clinical Science, University of Bergen, Bergen, Norway; 5 Department of Geriatric Medicine, Oslo University Hospital, 0424 Oslo, Norway; 6 Oslo Delirium Research Group, Department of Geriatric Medicine, Oslo University Hospital, 0424 Oslo, Norway

**Keywords:** Sepsis, qSOFA, Delirium, Infection, SOFA, Emergency department, 4AT, Encephalopathy

## Abstract

**Purpose:**

We aimed to study the use of The 4 ‘A’s test (4AT), a rapid delirium screening tool, performed upon Emergency Department (ED) admission, and to characterize older patients admitted to the ED with and without sepsis in terms of delirium features.

**Methods:**

In this prospective cohort study, we included patients aged ≥ 65 years, admitted to the ED with suspected sepsis. ED nurses and doctors performed delirium screening with 4AT within two hours after ED admission, and registered the time spent on the screening in each case. Sepsis and delirium during the hospital stay were diagnosed retrospectively, according to recommended diagnosis criteria.

**Results:**

Out of the 196 patients included (mean age 81 years, 60% men), 100 patients fulfilled the sepsis diagnosis criteria. The mean 4AT screening time was 2.5 Minutes. In total, 114 patients (58%) had a 4AT score ≥ 1, indicating cognitive impairment, upon ED admission. Sepsis patients more often had a 4AT score ≥ 4, indicating delirium, than patients without sepsis (40% vs. 26%, *p* < 0.05). Out of the 100 patients with sepsis, 68 (68%) had delirium during the hospital stay, as compared to 34 out of 96 patients (35%) without sepsis (*p* < 0.05).

**Conclusion:**

Delirium screening upon ED admission, using 4AT, was feasible among patients aged ≥ 65 years admitted with suspected sepsis. Two out of three patients had at least one feature of delirium upon admission. The prevalence of delirium during the hospital stay was high, particularly in patients with sepsis.

**Graphic abstract:**

**Supplementary Information:**

The online version contains supplementary material available at 10.1007/s41999-021-00558-5.

## Key summary points

**Aim:**

To study delirium screening upon Emergency Department admission among patients admitted with suspected sepsis.

**Findings:**

Delirium screening upon Emergency Department admission, using 4AT, was useful among patients aged ≥65 years admitted with suspected sepsis. Two out of three patients had at least one feature of delirium upon admission.

**Message:**

This study suggest increased awareness of delirium among older patients with suspected sepsis.

**Supplementary Information:**

The online version contains supplementary material available at 10.1007/s41999-021-00558-5.

## Graphical abstract

**Figure:**
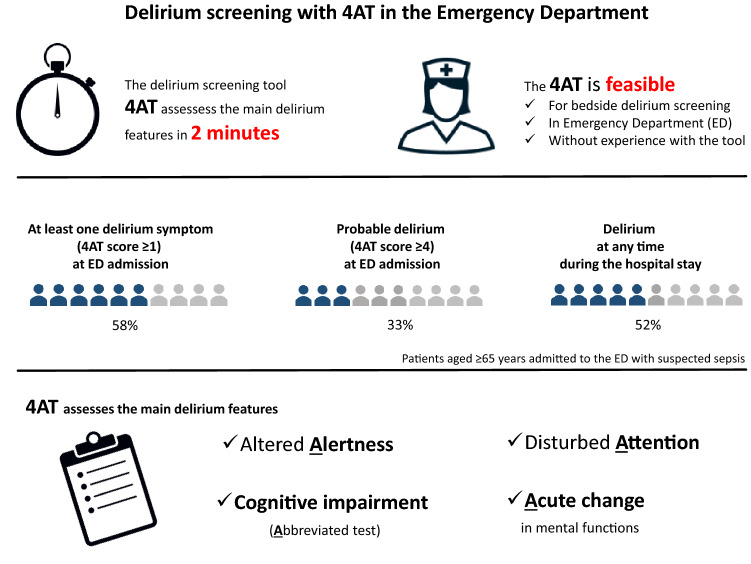
Delirium screening with 4AT in the Emergency Department

## Introduction

Sepsis is severe organ dysfunction caused by a dysregulated host response to infection [1], and responsible for one out of five deaths worldwide [2]. Older people are more susceptible to severe infections and sepsis, probably due to multiple factors, such as comorbidities, reduced physiological organ reserves, attenuated immune function, and institutionalization [3, 4]. Yet, few studies have characterized old patients with sepsis in detail. Older people with acute illness more often present with discrete or atypical symptoms than younger, often leading to delayed diagnosis and treatment [5]. Cognitive impairment and delirium are highly prevalent among acutely ill older people, and might further complicate the diagnostic evaluation. The incidence of delirium increases with age [6, 7, 8,9], and infections are among the most common precipitating factors [9, 10]. Delirium is an encephalopathy characterized by an acute alteration in alertness and attention, with additional disturbances in cognition (i.e., memory deficit, disorientation, perception or language) that develops over a short period of time [11]. Delirium can be an important sign of clinical deterioration, and should prompt further evaluation and treatment of underlying causes. Furthermore, delirium is associated with increased mortality. Early recognition of delirium is important, because treatment can improve outcomes and general patient care. Although at least one out of ten patients admitted to Emergency Departments (EDs) are suffering from delirium [10], screening for this clinical entity is not performed routinely in many EDs, and delirium often remains undetected [12].

Clinical risk scores are extensively used in EDs to facilitate optimal management of patients. The quick Sequential Organ Failure Assessment (qSOFA) is a recommended tool to predict poor outcomes in patients with infection [1]. Altered mental status is one of three clinical variables scored in the qSOFA. Thus, evaluation of cognitive function is now a core component of emergency medicine. However, the qSOFA only offers a crude evaluation of cognitive function. The 4 ‘A’s Test (4AT) is a rapid bedside delirium screening tool that assesses delirium’s major features; disturbed alertness, cognition and attention, and acute change or fluctuation in symptoms (https://www.the4at.com/) [13]*.* The 4AT has high sensitivity and specificity to detect delirium, and has been validated for use among ED patients [12]. However, we are not aware of any studies that have evaluated the feasibility of the 4AT upon ED arrival for suspected sepsis, performed by nurses and doctors without any previous experience with this tool.

In this study, we aimed to investigate the feasibility of 4AT performed in older patients upon admission to the ED with suspected sepsis, and the incidence of cognitive impairment and delirium among them.

## Methods

In this prospective cohort study, we consecutively included patients aged 65 years and older with suspected sepsis (as judged by ED nurses or doctors), admitted to the EDs of two Norwegian general hospitals; Bærum Hospital Vestre Viken Hospital Trust in the Oslo region, and Haraldsplass Deaconess Hospital in Bergen, western Norway. Bærum Hospital is one of the largest regional hospitals in Norway with a catchment area of approximately 200 000 inhabitants. More than 22,000 patientes are admitted to the ED yearly. Out of these, around 9500 are treated in the Department of Internal Medicine. The catchment area for Haraldsplass Deconess Hospital is approximately 145,000 inhabitants. In 2018, around 9300 patients were admitted to the Department of Medicine, of whom 8250 were 65 years or older. For practical reasons, patients were included during the periods from October 23rd 2017 to May 14th 2018 at Bærum Hospital, and June 18th to October 14th 2018 at Haraldsplass Deaconess Hospital. Patients for whom an International Classification of Diseases 10th Revision (ICD-10) code corresponding to an infectious disease was not registered for the current hospital stay, were excluded from the analysis. A list of the infectious disease ICD-10-codes used for this purpose, is provided as Supplementary material (Supplementary table 1).

### Assessments

***4AT*** The 4 ‘A’s test assesses four core features of delirium [13]. A total 4AT score of 1–3 indicates possible cognitive impairment, and a score of four or above indicates possible delirium. In patients with a 4AT score of 0, delirium or severe cognitive impairment are unlikely. The 4AT consists of four subscores, each representing one of the four ‘A’s; Alertness, Abbreviated Mental Test-4 (AMT4), Attention, and Acute change or fluctuating course: (1) *Alertness* (Is the patient fully alert and not markedly drowsy or agitated?). Patients with altered alertness during bedside assessment, are scored with four points. Patients who are not agitated, and fully alert, or have mild sleepiness for less than 10 s, are scored with 0 points. (2) *AMT4* This abbreviated cognitive assessment tests if the patient is oriented. The patient is asked to tell her/his age, date of birth, the name of the hospital or building, and the current year. While one mistake is scored with one point, and two or more mistakes with two points, correct answers give a score of 0. (3) *Attention* The patient is asked to list the months of the year in backward order. Patients who manage less than seven months are scored with one point. Patients who are not testable due to drowsiness or disturbed attention, are scored with two points. Patients managing seven or more months correctly, are scored with 0. (4) *Acute change or fluctuating course* If there is evidence of change or fluctuation in alertness, cognition or other mental functions that have arisen over the last two weeks and are still present within the last 24 h, the patient is scored with four points. If not, the patient is scored with 0 points. This subscore often requires information from a next of kin. When performed by delirium experts or researchers, the 4AT detects delirium in acutely ill patients with a sensitivity of 76% and a specificity of 95% [12]. We used the Norwegian translation of the 4AT in this study [14]. 4AT was conducted by ED nurses and doctors without any previous experience with the screening tool, except for a 45-min introduction lecture. The screening was performed in the ED within two hours of admission. The operating nurse or doctor registered the number of minutes used on the delirium screening, and if they found the screening tool useful or not in each case.

***qSOFA*** The qSOFA score assesses the risk of poor outcomes in patients with infections. Systolic blood pressure ≤ 100 mmHg, respiratory rate of ≥ 22/min, and altered mental status are scored with one point each. The most recent consensus on sepsis management (Sepsis-3) suggests that one point for altered mental status should be given when the Glasgow Coma Scale score is < 15 [1]. A qSOFA score ≥ 2 should prompt clinicians to further investigate for organ dysfunction, initiate sepsis therapy, and consider increased monitoring.

***Delirium*** We diagnosed delirium retrospectively by a thorough review of the patients’ hospital records, and according to the Diagnostic and Statistical Manual of Mental Disorders (DSM) 5 [15]. We used a chart-based method aiming to extract evidence for each of the diagnostic criteria, from hospital records, including daily notes by both doctors, nurses, and other staff. This method has been used in previous studies [16], and shows acceptable validity when performed by delirium experts or delirium researchers [17, 18]. We classified cases with evidence of an acute change in alertness, cognition, or other mental functions, as delirium [11].

***Sepsis*** We defined sepsis according to existing guidelines, as an evident infection, based on the history and a thorough clinical evaluation, along with laboratory and radiological findings, with an acute (within 24 h) change in total Sequential Organ Failure Assessment (SOFA) score of ≥ 2 points consequent to the infection [1]. SOFA grades impairment by organ system in patients with infection, and accounts for clinical interventions [19]. A change in SOFA score ≥ 2 points indicates organ dysfunction [20]. Septic shock was defined as sepsis identified by SOFA with a mean arterial pressure less than 65 mmHg despite vasopressor therapy, and hyperlactatemia (> 2 mMol/L, 18 mg/dL) after volume resuscitation [1]. We also registered ICD-10 codes for sepsis (A39.2, A40, A41, I33.0, G00, R65) registered at discharge from the hospital.

***Infection diagnoses*** Infections were defined based on the ICD-10 codes from the actual hospital stay.

***Mortality*** In-hospital mortality was defined as death from any cause during the hospital stay.

***Other measures*** Age, length of hospital stay, and discharge destination were retrieved from hospital records. Hospital stay was defined as the period from admission to the ED, to discharge from hospital.

### Statistical methods

Continuous variables are presented as the mean ± standard deviation and categorical variables as a number (%). We used Student’s *t* test for means of continuous variables, and Pearson’s Chi-square test of independence for categorical variables, to compare characteristics between patients with and without sepsis, different age groups, and study site. A *p* value < 0.05 was considered statistically significant. All statistical analyses were conducted using SPSS version 25.0 (IBM, Armonk, NY, USA).

### Patient and public involvement

Patient or public involvement in the design, execution or dissemination of results of the present study was not considered feasible or relevant.

## Results

**Fig. 1:**
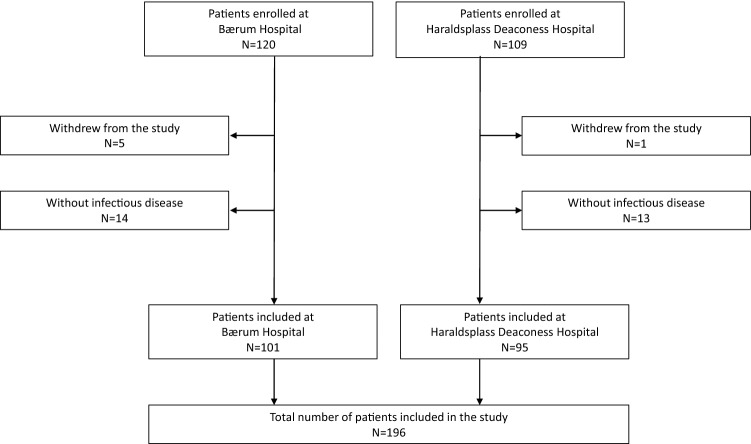
Flow chart illustrating the inclusion of patients at Bærum Hospital and Haraldsplass Deaconess Hospital in the study

A total of 229 patients aged 65 years and older were enrolled in the study. After excluding six patients who withdrew from the study, and 27 patients without an infectious disease (as defined by ICD-10 codes), 196 patients, with a mean age of 81.1 years, were eligible for further analysis (101 at Bærum Hospital and 95 at Haraldsplass Deaconess Hospital, respectively). 117 (60%) were men. Figure 1 shows a flow chart illustrating the inclusion in the study. Characteristics of the study participants by study hospital are shown in Supplementary table 2. Pneumonia was the most common diagnosis (45%), followed by urinary tract infection (34%), influenza (8%), skin and soft tissue infections (6%), abdominal infections (5%), and others (7%).

In total, 100 patients (51%) fulfilled the sepsis criteria, of whom three had septic shock. Table 1 shows the characteristics of patients with and without sepsis. Those with sepsis were more likely to be men, and more often had renal failure and anemia, than patients without sepsis. Only 37 out of 100 patients (37%) were discharged with an ICD-code of sepsis.

**Table 1:** Characteristics of patients aged 65 years and older admitted to two Norwegian Emergency Departments, by sepsis diagnosis (*n* = 196)

	Sepsis (*n* = 100)	No sepsis (*n* = 96)	*p* value
	Mean (SD)	Mean (SD)	
Age (years)	81.3 (7.8)	80.9 (7.9)	0.75
Length of hospital stay (days)	8.1 (6.3)	6.9 (6.5)	0.18
C-reactive protein (mg/L)^a^	177 (98)	153 (104)	0.10

	*n* (%)	*n* (%)	
Men	67 (67)	50 (52)	< 0.05
Age group (years)			0.85
65–79	42 (42)	39 (41)	
≥ 80	58 (58)	57 (59)	
Renal function (mL/min/1.73 m^2^)			< 0.05
eGFR > 60	36 (36)	56 (56)	
eGFR < 30	24 (24)	5 (5)	
Hyponatremia^b^			0.59
Mild	30 (31)	26 (27)	
Moderate	4 (4)	2 (2)	
Anemia^c^			< 0.05
Moderate	83 (83)	66 (69)	
Severe	7 (7)	3 (3)	
Infection diagnosis^d^			
Pneumonia	41 (41)	46 (48)	0.33
Urinary tract infection	24 (25)	31 (32)	0.20
Influenza	9 (9)	6 (6)	0.47
Abdominal infection	7 (7)	3 (3)	0.22
Skin infection	5 (5)	7 (7)	0.50
Others	8 (8)	6 (6)	0.63

*SD* standard deviation, *eGFR* estimated Glomerular Filtration Rate

^a^Maximum value during the hospital stay

^b^Mild hyponatremia, serum sodium 130–136 Mmol/L; moderate hyponatremia, serum sodium 120–129 Mmol/L

^c^Moderate anemia, hemoglobin < 12 g/dL for women, and < 13 g/dL for men; severe anemia, hemoglobin < 8 g/dL

^d^Based on International Classification of Diseases-10 codes

The median time spent on delirium screening with 4AT was two minutes (mean 2.5 min, registered in 164 (84%) patients). While qSOFA identified 48 patients with altered mental status, 114 patients (58%) had a 4AT score of at least one upon ED admission, indicating cognitive impairment. The operators valued the 4AT screening as useful in 77 out of 89 cases (78%) with cognitive impairment, and 43 out 68 cases (63%) without cognitive impairment (not reported in 30 cases). Table 2 shows qSOFA and 4AT scores in patients with and without sepsis. Sepsis patients more often had a 4AT score ≥ 4 upon ED admission, indicating delirium, than patients without sepsis (40% vs. 26%, *p* < 0.05). Disturbed attention (assessed with the Months Backwards Test) was the most common finding.

**Table 2:** Quick Sequential Organ Failure Assessment scores and 4AT scores at Emergency Department admission by sepsis diagnosis in patients aged 65 and older admitted to two Norwegian Emergency Departments with suspected sepsis (*n* = 196)

	Sepsis (*n* = 100)	No sepsis (*n* = 96)	*p* value
	Mean (SD)	Mean (SD)	
qSOFA score^a^			< 0.05
0	11 (11)	25 (26)	
1	42 (42)	48 (50)	
2	36 (36)	18 (19)	
3	8 (8)	0 (0)	
Respiratory rate ≥ 22/min	81 (81)	56 (58)	< 0.05
Systolic BP ≤ 100 mmHg	28 (28)	16 (17)	0.06
Altered mental status (GCS < 15)^a^	31 (31)	17 (18)	0.08
4AT score^b^			< 0.05
0	31 (31)	49 (52)	
1–3	29 (29)	21 (22)	
≥ 4	40 (40)	24 (26)	
4AT subscores			
Reduced alertness	19 (19)	12 (13)	0.17
Cognitive impairment	56 (56)	27 (28)	< 0.05
Disturbed attention	59 (59)	38 (40)	< 0.05
Acute change or fluctuations	28 (28)	19 (21)	0.12

*SD* standard deviation, *SOFA* Sequential Organ Failure Assessment, *GCS* Glasgow Coma Scale, *BP* blood pressure. *4AT* 4 Assessment Test

^a^Altered mental status scored with one point when Glasgow Coma Scale score was < 15. Glasgow Coma Scale score and Quick Sequential Organ Failure Assessment score were missing in three patients with sepsis and five patients without sepsis

^b^4AT score was missing in two patients without sepsis

Characteristics, qSOFA score, and 4AT score in sepsis patients aged under 80 years and 80 years and older are shown in Supplementary table 3. Although not statistically significant, older sepsis patients more often had symptoms of cognitive impairment at admission than younger sepsis patients.

A total of 102 patients (52%) had delirium anytime during the hospital stay. Out of these, 61 (60%) had a 4AT score ≥ 4, indicating delirium upon ED admission. The prevalence of delirium anytime during the hospital stay was 44% in patients aged 65–80 years, and 57% in patients aged 80 years and older. Out of the 100 patients with sepsis, 68 (68%) had delirium during the hospital stay, compared to 34 out of 96 patients (35%) without sepsis (*p* < 0.05). Patients aged 80 years and older with sepsis had the highest delirium prevalence (44 out of 58; 76%).

Ten patients (5%) died during hospital stay; nine patients with sepsis and one patient without sepsis (in-hospital mortality 9% vs. 1%, *p* < 0.05). In-hospital mortality was 14% in sepsis patients aged ≥ 80 years and 2% in sepsis patients aged 65–79 (*p* < 0.05).

## Discussion

To our knowledge, this is the first study evaluating the feasibility of delirium screening with 4AT upon ED admission carried out by nurses and doctors without any previous experience with this screening tool. The use of 4AT was feasible, and compared with qSOFA, gave a more detailed characterization of cognitive impairment and features of delirium. In line with previous studies of acutely ill older patients, we found that cognitive impairment or delirium symptoms were highly prevalent already at hospital admission [6–9].

In particular, many patients with sepsis had a 4AT-score of four or more, indicating delirium. Delirium features can represent clinical deterioration, and is associated with poor outcomes [9]. The pathophysiological mechanisms explaining delirium in acutely ill patients are not fully understood [21]. However, in patients with sepsis, both cerebral hypoperfusion due to hypotension, and cerebral hypoxia, could play a role.

This study demonstrated a particularly high prevalence of delirium among patients with sepsis aged 80 years and older. It has been suggested that delirium screening should be implemented in routine practice in patients groups with a high risk of delirium [7, 12]. Our findings support that increased awareness of delirium in older patients acutely admitted to hospital with suspected sepsis is necessary, and indicate a high prevalence of delirium features already at ED admission. We believe that systematic screening is useful in this patient group, but this needs to be addressed in future studies.

The 4AT has been characterized as a rapid and practical tool that does not require special training. Validation studies of 4AT have demonstrated high sensitivity and specificity to predict delirium among ED patients [12, 22, 23]. However, in these studies, 4AT has been performed by delirium experts or trained research assistants, often many hours after hospital admission. The validity of the 4AT might be lower in other settings and dependent on both the operators and the assessment timing [17]. Hence, further studies are needed to explore its use in different settings.

The risk of delayed sepsis treatment and the high workload in many EDs could be counterarguments to introducing a new diagnostic tool. Nevertheless, in patients with cognitive impairment or delirium, rapid screening with 4AT might provide important additional information. In this study, the median time spent on the 4AT assessment was only two minutes, and in line with a previous report, nurses and doctors valued delirium screening as useful in the majority of the cases [12].

Although the mean age of the patients in the current study was higher than in previous studies, the overall in-hospital mortality rate of 5% corresponds well with a Norwegian study of sepsis patients admitted to the ED of a university hospital, where the mortality after seven days was 7% [24].

### Strengths and limitations

A strength of our study is the inclusion of old patients with symptoms of cognitive impairment and delirium; patient groups that are often excluded from clinical studies. Furthermore, we investigated a delirium screening tool used upon ED admission, performed by nurses and doctors inexperienced with the tool, and without particular expertise in delirium diagnosis. Such an approach, together with the inclusion of patients at two general hospitals in different cities, might have improved our findings’ generalizability. On the other hand, differences in patient inclusion might have introduced skewness in the data, with the risk of bias. While the inclusion took place between October 2017 and May 2018 at Bærum Hospital, patients were included from June to October 2018 at Haraldsplass Deaconess Hospital, and seasonal differences in the prevalence of infections may have had an impact on the results. Further limitations were the retrospective registration of sepsis and delirium diagnoses, the lack of information about comorbidities, frailty, and medication, and the lack of information about patients excluded from the study. Furthermore, the Norwegian translation of the 4AT has not been validated. And finally, the study did not assess interoperator variability, and the limited operator training, and many operators involved in the study could have biased the results.

In conclusion, the delirium screening tool 4AT was useful upon ED admission in patients aged 65 years and older with suspected sepsis, when performed by nurses and doctors inexperienced with this tool. Two out of three patients had at least one feature of delirium upon ED admission. Sepsis patients more often had a 4AT score ≥ 4, indicating delirium. More than half of the patients had delirium during the hospital stay, and delirium was more common among patients with sepsis. Our findings suggest that increased awareness of delirium features among older patients with suspected sepsis is important. Future studies should further evaluate the value of delirium screening with 4AT in different settings.

## Supplementary Information

Below is the link to the electronic supplementary material.

Supplementary file1 (DOCX 12 kb) Supplementary table 1. Infectious disease Classification of Diseases 10th Revision (ICD-10) codes used to identify patients eligible for the analysis

Supplementary file2 (DOCX 19 kb) Supplementary table 2. Characteristics of included patients (n = 196) by study site

Supplementary file3 (DOCX 14 kb) Supplementary table 3. Characteristics of patients with sepsis by age group (n = 100), among patients aged 65 and older admitted to two Norwegian Emergency Departments with suspected sepsis

## Declarations

### Availability of data and materials

The datasets generated and/or analyzed in the current study are not publicly available, but available from the corresponding author upon reasonable request.
