# Supplementary material for: Delirium screening with 4AT in patients aged 65 years and older admitted to the Emergency Department with suspected sepsis: a prospective cohort study
Source: Eur Geriatr Med. 2021 Oct 8;13(1):155–62. doi: 10.1007/s41999-021-00558-5 (PMC8860779; doi:10.1007/s41999-021-00558-5)
Supplement: Supplementary file 1 — Supplementary file1 (DOCX 12 kb) Supplementary table 1. Infectious disease Classification of Diseases 10th Revision (ICD-10) codes used to identify patients eligible for the analysis [file 41999_2021_558_MOESM1_ESM.docx]

**Supplementary table 1.** Infectious disease Classification of Diseases 10th Revision (ICD-10) codes used to identify patients eligible for the analysis.

| A02 |
| --- |
| A04 |
| A20 |
| A21 |
| A22 |
| A24 |
| A26 |
| A32 |
| A39 |
| A40 |
| A41 |
| A42 |
| A46 |
| A48 |
| A54 |
| B00 |
| B01 |
| B02 |
| B37 |
| B96 |
| G00 |
| I33 |
| J01 |
| J03 |
| J06 |
| J09 |
| J10 |
| J13 |
| J14 |
| J15 |
| J15 |
| J15 |
| J18 |
| J22 |
| J36 |
| J39 |
| J44 |
| J85 |
| J85 |
| J86 |
| J86 |
| K65 |
| K80 |
| K80 |
| K81 |
| K81 |
| K83 |
| L03 |
| M00 |
| M46 |
| M72 |
| M86 |
| N10 |
| N30 |
| N30 |
| N39 |
| N41 |
| N45 |
| R57 |
| R65 |
| T81 |
| T82 |
