# Supplementary material for: Delirium screening with 4AT in patients aged 65 years and older admitted to the Emergency Department with suspected sepsis: a prospective cohort study
Source: Eur Geriatr Med. 2021 Oct 8;13(1):155–62. doi: 10.1007/s41999-021-00558-5 (PMC8860779; doi:10.1007/s41999-021-00558-5)
Supplement: Supplementary file 2 — Supplementary file2 (DOCX 19 kb) Supplementary table 2. Characteristics of included patients (n = 196) by study site [file 41999_2021_558_MOESM2_ESM.docx]

**Supplementary table 2.** Characteristics of included patients (n=196) by study site.

|  | **Haraldsplass**  **(n=95)** | **Bærum**  **(n=101)** | **p-value** |
| --- | --- | --- | --- |
|  | **Mean (SD)** | **Mean (SD)** |  |
| Age (years) | 81.1 (7.2) | 81.2 (8.4) | 0.95 |
| Length of hospital stay (days) | 7.2 (6.9) | 7.7 (5.9) | 0.56 |
| C-reactive Protein (mg/L)^1^ | 166 (100) | 164 (102) | 0.89 |
|  |  |  |  |
|  | **n (%)** | **n (%)** |  |
| Women | 38 (40) | 41 (41) | 0.93 |
| qSOFA ≥2 | 31 (33) | 33 (31) | 0.92 |
| Respiratory rate >22/min | 64 (67) | 73 (72) | 0.45 |
| Systolic BP <100 mmHg | 16 (17) | 28 (28) | 0.07 |
| Altered mental status (GCS≤14) | 29 (31) | 19 (20) | 0.09 |
| Renal function (mL/min/1.73 m^2^)^2^ |  |  | 0.22 |
| eGFR >60 | 38 (40) | 52 (52) |  |
| eGFR <30 | 15 (16) | 14 (14) |  |
| Hyponatremia^2^ |  |  | 0.54 |
| Mild | 29 (31) | 27 (27) |  |
| Moderate | 4 (4) | 2 (2) |  |
| Anemia^2^ |  |  | 0.62 |
| Moderate | 73 (77) | 76 (75) |  |
| Severe | 7 (7) | 4 (4) |  |
| 4AT-score |  |  | 0.61 |
| 0 | 38 (40) | 42 (42) |  |
| 1-3 | 22 (23) | 28 (28) |  |
| ≥4 | 35 (37) | 31 (31) |  |
| Reduced alertness | 15 (16) | 16 (16) | 0.39 |
| Cognitive impairment | 48 (50) | 35 (34) | 0.05 |
| Disturbed attention | 48 (50) | 49 (47) | 0.40 |
| Acute change or fluctuations | 29 (32) | 18 (18) | 0.06 |
| Infection diagnosis^3^ |  |  |  |
| Pneumonia | 42 (44) | 45 (45) | 0.96 |
| Urinary tract infection | 33 (35) | 22 (22) | 0.05 |
| Influenza | 0 (0) | 15 (15) | <0.05 |
| Abdominal infection | 5 (5) | 5 (5) | 0.92 |
| Skin infection | 5 (5) | 7 (7) | 0.63 |
| Others | 11 (12) | 3 (3) | <0.05 |
| Infection and ≥2 SIRS criteria | 71 (73) | 75 (74) | 0.94 |
| Sepsis | 45 (47) | 55 (55) | 0.32 |
| Septic shock | 2 (2) | 1 (1) | 0.54 |
| ≥5 drugs at discharge | 77 (81) | 73 (72) | 0.07 |
| Discharge destination |  |  | 0.22 |
| Home | 47 (50) | 54 (54) |  |
| Institution | 39 (41) | 42 (42) |  |
| In-hospital mortality | 5 (5) | 5 (5) | 0.92 |
| 28-day mortality | 7 (7) | 9 (9) | 0.69 |

^1^ Highest value during the hospital stay, ^2^ Lowest value during the hospital stay, ^3^ Based on International Classification of Diseases-10 codes.
SD, Standard Deviation; SOFA, Sequential Organ Failure Assessment; qSOFA, quick SOFA; GCS, Glasgow Coma Scale; BP, Blood Pressure; eGFR, estimated Glomerular Filtration Rate; 4AT, 4 Assessment Test; SIRS, Systemic Inflammatory Response Syndrom; Mild hyponatremia, Serum Sodium 130-136 Mmol/L; Moderate hyponatremia, Serum Sodium 120-129 Mmol/L.
