# Supplementary material for: Delirium screening with 4AT in patients aged 65 years and older admitted to the Emergency Department with suspected sepsis: a prospective cohort study
Source: Eur Geriatr Med. 2021 Oct 8;13(1):155–62. doi: 10.1007/s41999-021-00558-5 (PMC8860779; doi:10.1007/s41999-021-00558-5)
Supplement: Supplementary file 3 — Supplementary file3 (DOCX 14 kb) Supplementary table 3. Characteristics of patients with sepsis by age group (n = 100), among patients aged 65 and older admitted to two Norwegian Emergency Departments with suspected sepsis [file 41999_2021_558_MOESM3_ESM.docx]

**Supplementary table 3.** Characteristics of patients with sepsis by age group (n=100), among patients aged 65 and older admitted to two Norwegian Emergency Departments with suspected sepsis.

|  | **Age ≥80 years**  **(n=58)** | **Age <80 years**  **(n=42)** | **p-value** |
| --- | --- | --- | --- |
|  | **Mean (SD)** | **Mean (SD)** |  |
| Age (years) | 86.9 (4.6) | 73.6 (3.7) | <0.05 |
| Length of hospital stay (days) | 8.4 (7.6) | 7.6 (4.0) | 0.48 |
| C-reactive Protein (mg/L)^1^ | 164 (95) | 193 (100) | 0.15 |
|  |  |  |  |
|  | **n (%)** | **n (%)** |  |
| Men | 37 (64) | 30 (71) | 0.42 |
| qSOFA ≥2 | 25 (46) | 19 (45) | 0.98 |
| Respiratory rate >22/min | 48 (83) | 33 (79) | 0.60 |
| Systolic BP <100 mmHg | 17 (29) | 11 (26) | 0.73 |
| Altered mental status (GCS≤14) | 17 (31) | 14 (33) | 0.32 |
| Renal function (mL/min/1.73 m^2^)^2^ |  |  | <0.05 |
| eGFR >60 | 12 (21) | 24 (57) |  |
| eGFR <30 | 21 (36) | 3 (7) |  |
| Hyponatremia^2, 3^ |  |  | 0.09 |
| Mild | 12 (21) | 18 (43) |  |
| Moderate | 2 (3) | 2 (5) |  |
| Anemia^2, 4^ |  |  | 0.68 |
| Moderate | 48 (83) | 35 (83) |  |
| Severe | 5 (9) | 2 (5) |  |
| 4AT-score |  |  | 0.37 |
| 0 | 15 (26) | 16 (38) |  |
| 1-3 | 17 (29) | 12 (29) |  |
| ≥4 | 26 (45) | 14 (33) |  |
| 4AT subscores |  |  |  |
| Reduced alertness | 11 (19) | 8 (19) | 0.99 |
| Cognitive impairment | 37 (64) | 19 (45) | 0.11 |
| Disturbed attention | 37 (64) | 24 (52) | 0.33 |
| Acute change or fluctuations | 19 (33) | 9 (21) | 0.30 |
| Infection diagnosis^5^ |  |  |  |
| Pneumonia | 18 (31) | 23 (55) | <0.05 |
| Urinary tract infection | 15 (26) | 9 (21) | 0.61 |
| Influenza | 6 (10) | 3 (7) | 0.58 |
| Abdominal infection | 6 (10) | 1 (2) | 0.12 |
| Skin infection | 3 (5) | 2 (5) | 0.93 |
| Others | 6 (10) | 2 (5) | 0.31 |
| Discharge destination |  |  | <0.05 |
| Home | 20 (35) | 28 (67) |  |
| Institution | 29 (50) | 11 (26) |  |
| In-hospital mortality | 8 (14) | 1 (2) | <0.05 |

^1^ Highest value during the hospital stay, ^2^ Lowest value during the hospital stay, ^3^ Mild hyponatremia, Serum Sodium 130-136 Mmol/L; Moderate hyponatremia, Serum Sodium 120-129 Mmol/L, ^4^ Moderate anemia, Hemoglobin <12 g/dL for women and <13 g/dL for men ; severe anemia, Hemoglobin <8 g/dL, ^5^ Based on International Classification of Diseases-10 codes.
SD, Standard Deviation; SOFA, Sequential Organ Failure Assessment; qSOFA, quick SOFA; GCS, Glasgow Coma Scale; BP, Blood Pressure; eGFR, estimated Glomerular Filtration Rate; 4AT, 4 Assessment Test; SIRS, Systemic Inflammatory Response Syndrom; Mild hyponatremia, Serum Sodium 130-136 Mmol/L; Moderate hyponatremia, Serum Sodium 120-129 Mmol/L.
